# Iron as a Remedy

**Published:** 1903-03

**Authors:** 


### ABSTRACTS.

### Iron as a Remedy.

Time out of mind (*Medical Mirror*, November, 1902,) iron has been leaned upon, as one of the special standbys in medicine, particularly as a builder and reconstructor. But unless iron be given in proper form, one might as well give absorbent cotton, or chips or wet stones. When we desire to produce any increase in the number of red blood corpuscles, and to make them redder and richer with hemoglobin, we need to be sure of the form of iron that we are giving. The evidence has been accumulating these many years, that manganese, in itself an admirable remedy, combined with iron emphasises the potency of both.

Dr. Gude, the great German chemist, contributed very definitely to the good of the profession, when he presented the product of long years of experimentation and clinical experience, the therapeutic product known as pepto-mangan (Gude).

Added to the many hundreds of clinical contributions, Dr. J. W. Frieser, of Vienna, Austria, recently reports most favorably, and very forcibly, observing as follows:

Pepto-mangan contains iron and manganese combined with peptone in the proper proportions, and in a readily digestible and absorbable form, so that the preparation can be completely utilised by the organism. As is well known, the peptones represent artificial predigested products, which when taken into the organism make no special demands upon the digestive functions, which in anemic and chlorotic persons are usually weakened and impaired in action. This fact is the more important, since in these cases, the digestive process and the secretion of gastric juice is usually reduced, in consequence of which the nutrition is quite impaired, while frequently there is a condition of hyperacidity of the gastric juice. It has been most gratifying to me to observe that during the use of pepto-mangan (Gude), which experience has taught me is particularly adapted in these maladies, it does not interfere with, or exert any disturbing effect upon the digestion. On the contrary, under its administration, the appetite and the digestion are stimulated in a very satisfactory manner.

As a rule, during treatment with pepto-mangan the improvement in the constitution of the blood, as shown by physical examination, was accompanied by a beneficial effect upon the general condition and strength. The appearance and appetite of the patients improved visibly; the digestion and nutrition progressed favorably, and the patient felt better, happier and more vigorous.

